# Analysis of KRAS gene mutation associated with *Helicobacter pylori* infection in patients with gastric cancer

**DOI:** 10.22038/ijbms.2019.32047.7707

**Published:** 2019-05

**Authors:** Raheleh Jabini, Seyed Ahmad Eghbali, Hossein Ayatollahi, Maryam Sheikhi, Mohammadreza Farzanehfar

**Affiliations:** 1 Biotechnology Research Center, Pharmaceutical technology institute, Mashhad University of Medical Sciences, Mashhad, Iran; 2 Gastroenterology and Hepatology Specialist, Mashhad, Iran; 3 Pathology Department of Hematology and Blood Banking, Cancer Molecular Pathology Research Center, Mashhad University of Medical Sciences, Mashhad, Iran; 4 Gastroenterology and Hepatology Research Center, Mashhad University of Medical Sciences, Mashhad, Iran

**Keywords:** Helicobacter pylori, Gastric cancer, KRAS, Mutation, Sequencing

## Abstract

**Objective(s):**

KRAS proto-oncogene mutation can be considered a diagnostic factor for treating various malignancies. *Helicobacter pylori* infection, a risk factor for stomach cancer, may cause DNA damage and genetic changes. The aim of the current study was to assess the association of gastric cancer and KRAS mutation, demographic factors, and *H. pylori* infection.

**Materials and Methods:**

DNA was extracted from a total of 140 FFPE gastric cancer tissue samples. detection of KRAS mutation (codons 12 and 13) in tumors was performed by PCR amplification, followed by gel electrophoresis and DNA sequencing. PCR diagnosed any *H. pylori* infection.

**Results:**

KRAS mutation was detected in 6 of the 140 (4.2%) gastric cancer tissue samples. 18 samples (12.8%), all of which were male (*P<*0.05), tested positive for *H. pylori* infection. KRAS mutations were present in 22.2% (4/18) of the samples with H. pylori infection (*P*<0.05). The mean age of patients was 62.25±12.61 years (range: 30–93 years). A male predominance (2.5 to 1) was reported in the gastric cancers, and at diagnosis, women were significantly younger than men (P=0.004). No association was observed between age or gender and KRAS mutation. Neither was one found between age and *H. pylori* infection. Tumors from *H. pylori*^+^ subjects were significantly more likely to have KRAS mutation than tumors from *H. pylori*^-^ subjects (OR=17.1).

**Conclusion:**

The data suggest that H. pylori infection when compared with the absence of *H. pylori* infection, is associated with a higher prevalence of KRAS mutation in gastric cancer.

## Introduction

Gastric cancer is one of the leading causes of death worldwide (1). Although age-standardized mortality rates have fallen constantly over the recent years even in high-risk countries, gastric cancer still accounts for a large percentage of cancer cases among both men and women in Asia, Latin America and some countries in Europe (2). Gastric cancer is the second common cancer in men and the fourth in women in Iran (3). Despite numerous studies, the molecular aspects underlying gastric cancer (GC) are not well understood. However, it has been shown that gastric cancer may be caused by DNA mutations that turn on oncogenes (4). The KRAS gene, an oncogene from the mammalian RAS gene family, encodes the KRAS protein, which is involved in multiple pathways, including proliferation, differentiation, and apoptosis (5). KRAS mutation in exon 2 leads to increased KRAS protein activity, which constitutively activates the mitogenic signal transduction pathway (4). Several studies have reported that KRAS point mutation is involved in the development of several types of cancer, such as pancreatic and colorectal cancers (6-9). In spite of much research, the clinicopathologic significance of KRAS mutation in gastric cancer remains unknown (4). According to recent studies, *Helicobacter pylori*, the main agent of chronic gastritis and ulcers, is a factor that may increase the risk of gastric cancer (10). Many prospective case-control studies have attempted to assess the link between *H. pylori* infection and development of gastric cancer, but they are inconsistent in their findings (11). In this study, we analyzed 140 FFPE samples of gastric cancer tissues for KRAS mutation and to detect *H. pylori* infection. The aim of the present study was to evaluate the relationship between KRAS gene mutation and *H. pylori* infection in gastric adenocarcinoma patients. 

## Materials and Methods

***Patients and Samples***

The current research conducted a review of gastric cancer patients who had undergone surgery between May 2001 and December 2010 at Ghaem Hospital of Mashhad University of Medical Sciences, Mashhad, Iran; available tissue blocks were obtained retrospectively. All samples were independently reviewed and tumor-rich areas were marked on hematoxylin and eosin slides by two experienced pathologists. This study was approved by the Ethics Committee of Mashhad University of Medical Sciences.

***DNA extraction from formalin-fixed paraffin embedded tissue***

Ten paraffin sections of 5µm were obtained from FFPE tissues and placed into a microcentrifuge tube. Deparaffinization and xylene removal methods were followed according to the standard protocol. According to the manufacturer’s protocol, DNA was extracted using a DNA extraction from tissue mini-kit (YT9030, Iran) and kept at -20 ^°^C. 

***PCR amplification and sequencing of KRAS***

DNA was amplified with a primer set flanking exon 2 (codons 12 and 13) of the KRAS gene (forward primer, 5’-GGTGAGTTTGTATTAAAAGGTACTGG-3’ and reverse primer 5’- TCCTGCACCAGTAATATGCA-3’). With the employment of the Taq DNA polymerase Master Mix RED (Amplicon; No. A180303), PCR was performed. After PCR, the samples were electrophoresed on 2% (w/v) agarose gel, stained with ethidium bromide solution, and visualized under UV light. PCR products were sequenced in both sense and antisense directions with the ABI 3130xl Genetic Analyzer (Applied Biosystems, Foster City, California, USA). Sequencing data were analyzed using the CLC Bio software (Qiagen, Boston, MA).

***Detection of H. pylori infection***

The *H. pylori* infection diagnosis was carried out using PCR and an *H. pylori* PCR detection Kit (Sinaclon; PR7843; Iran).

***Statistical analysis***

SPSS, version 21, was use to perform the statistical analysis. The Mann Whitney or chi-square test determined the correlation between the KRAS mutation and clinicopathologic parameters. *P-values* less than 0.05 were regarded as statistically significant.

## Results

**Figure 1:**
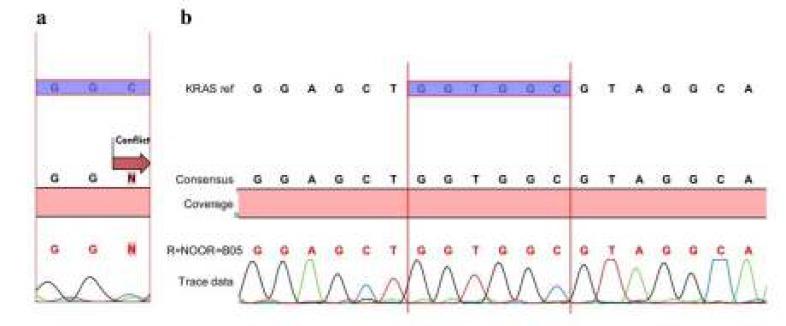
Sequencing analysis of the KRAS gene, mutant (a), wild type (b)

Patient data are presented in Table 1. Out of the current study^’^s140 non-recurrent GC samples, 100 were from males; males outnumbered females by a ratio of 2.5 to 1. The mean patient age at presentation was 62.25±12.61 years (age range: 30–93). All of the 140 patients were newly diagnosed with non-recurrent gastric cancer tumors. Women were significantly younger than the men at diagnosis: 57.75±10.55 versus 64.06±12.96 years (=0.004). Most of the patients were in their sixties. KRAS mutation was detected in 6 (4.2%) of the 140 patients (Figure1). Of the 6 patients with KRAS mutation, 3 were 60 or older and 3 were under 60 years of age. No association was found between the KRAS mutation and age or sex (Table 2). *H. pylori* positivity was reported at 12.8% (n=18). All 18 *H. pylori*-positive patients were male (Table 3, *P˂*0.05). As Table 3 indicates, *H. pylori* positivity was detected in 10 patients younger than 60 years and in 8 patients 60 years of age or above. No relationship was found between the presence of *H. pylori* infection and age. Table 4 presents a significant correlation between KRAS mutation and *H. pylori* infection (chi-square test, *P˂*0.05). According to Table 4, tumors from *H. pylori*^+^ subjects were significantly more likely to have KRAS mutation than tumors from *H. pylori*^-^ subjects (OR=17.1).

## Discussion

Stomach cancer continues to be one of the most prevalent cancers in the world (12). Gastric cancer is also known as a common fatal cancer in Iran (13). The most practical treatment options for gastric cancer are surgery, chemotherapy, and radiotherapy (14). However, some gastric cancer patients may fail to respond to various therapies. Despite much research, no standard chemotherapy regimen has been determined for gastric cancer patients (15). For the early detection and diagnosis of cancer, it is essential to study the etiology of how cancer develops. Recent studies have shown that the accumulation of genetic and epigenetic alterations convert a normal cell into a cancerous one (16-19). KRAS protein participates in the RAS-RAF-MAPK pathway and plays a key role in the control of proliferation and the survival of eukaryotic cells (20). It has been demonstrated that the RAS gene is commonly mutated in different types of human cancer (21). The KRAS gene is one of the first human oncogenes studied in cancer. It has also been demonstrated that the KRAS mutation status can be predictive of the response to drugs targeting the epidermal growth factor receptor (22). Lievre *et al.* have reported an association between the mutation status of KRAS and the clinical outcome of patients with colorectal cancer (23). Further studies are needed to understand the biological function of the KRAS mutation (24). *H. pylori* is a bacteria infecting approximately 50% of the world’s population (25). Chronic Gastritis, especially caused by *H. pylori*, may progress to gastric cancer as *H. pylori* may induce genetic changes in mucosal gastric cells (10). Although different invasive and non-invasive methods have been established to detect *H. pylori* infection, identifying *H. pylori*-infected patient is an ongoing challenge. In this study, we applied a PCR assay for the detection of *H. pylori* infection that is a highly specific (69%), sensitive (94%), and reproducible technique (26). Reflecting global statistics, gastric cancer in the current study was most commonly in patients aged between 50 and 60. In agreement with prior studies (27, 28), the present work’s results showed that males were predominantly diagnosed with gastric cancer (Table 1). This higher frequency in men may be explained by the greater number of men in high-risk occupations. In 1986, for the first time, a mutation in the KRAS gene of a gastric cancer patient was reported (29). In some studies, no KRAS mutation was found in the GC samples (30-33). While in three separate studies by Miki *et al.* (1991), Hongyo *et al.* (1995), and Corso *et al.* (2011), the KRAS gene was mutated in 13%, 21%, and 17% of the GC samples, respectively (the numbers of samples were 31, 34, and 63, respectively) (34-36). Considering genetic changes in cancers and the promise of cancer gene therapy, the current study assessed the mutation status of the KRAS gene in gastric cancer. There are several kinds of assays to evaluate KRAS mutation, of which many are based on PCR (37). In the present work, the screening and identification of gene mutation in tumors were performed by PCR amplification, followed by gel electrophoresis and DNA sequencing. Sequencing is a practical and powerful technique in molecular genetics. It confirms definitively the specificity of amplification (38). In the current study, the KRAS gene was found to be mutated in six of the samples (Figure 1; Table 4; 4.3 %, n=140). Based on the current article’s data, neither age nor sex was significantly correlated with KRAS mutation (Table 2). This finding is contrary to that of another study that reported a higher frequency of KRAS mutations among GC patients under 40 years of age (39). One possible reason for the variety of research results is the variety of stomach tumors. In the present work, adenocarcinoma tumors were examined. In addition, sample size and testing methods influence research outcomes. Unlike some other studies (40, 41), the present work’s results indicated that *H. pylori* infection correlated with the male gender as all 18 *H. pylori*-positive patients were male (Table 3, *P˂*0.05). This is in consonance with a study of adults in Canada, in which the overall prevalence of *H. pylori* infection was 23.1% but higher in men (29.4%) than in women (14.9%). One reason that has been suggested for the lower prevalence in women is that they may be more likely to clear *H. pylori* infection because of higher rates of incidental antibiotic use for other infectious diseases (42). In agreement with previous studies (43-44), the current study observed an association between KRAS gene mutation and *H. pylori* infection in gastric cancer patients (Table 4). According to the table shown, tumors from *H. pylori*^+^ subjects were significantly more likely to have KRAS mutation than tumors from *H. pylori*^-^ subjects (OR=17.1).

In other words, *H. pylori* infection leads to chronic inflammation and subsequent release of reactive radical compounds causing oxidative DNA damage and ultimately leading to cancer cells (45).

**Table 1:** Patient characteristics (total number of patients: n = 140, mean age: 62.25 ± 12.61 years, and age range: 30–93 years)

Sex	F	P%	Age (mean)	*P-value*
Female	40	28.6	57.75±10.55	P=0.04
Male	100	71.4	64.06±12.96	

F, frequency; P%, percent

**Table 2:** Demographic data of patients with and without KRAS mutation (n = 140)

Characteristic	KRAS mutation		*P*-value
	Positive	Negative	
Sex			
Female	2	38	
Male	4	96	*P*>0.05
Age			
<60	3	73	
≥60	3	61	*P*>0.05

**Table 3:** Demographic data of patients with and without *Helicobacter pylori* infection (n = 140)

Characteristic	*Helicobacter pylori* infection				*P*-value
	Positive		Negative		
	F	P%	F	P%	
Sex					
Female	0	0	40	28.57	
Male	18	12.85	82	58.57	*P*<0.05
Age					
<60	10	7.14	66	47.14	
≥60	8	5.71	56	40	*P*>0.05

F, frequency; P%, percent

**Table 4:** Odds ratio (OR) and correlation between *Helicobacter pylori* infection and KRAS gene mutation (n = 140)

	KRAS mutation		OR	*P-value*
	Positive	Negative		
*Helicobacter pylori* infection				
Positive	4	14	17.1	0.000
Negative	2	120		0.000
Total	6	134		

## Conclusion

We found KRAS mutation was more prevalent (17.1 times) in tumors from *H. pylori*^+^ patients than in tumors from *H. pylori*^-^ patients. The data of the present article demonstrated a significant association between *H. pylori* infection and KRAS gene mutation in gastric cancer patients. Additional studies are needed to expand the basic knowledge of the molecular pathology of gastric cancer and to investigate the prognostic value of its proto-oncogene.
